# Interaction between piperine and genes associated with sciatica and its mechanism based on molecular docking technology and network pharmacology

**DOI:** 10.1007/s11030-020-10055-9

**Published:** 2020-03-04

**Authors:** Jiu-wang Yu, Hong-wei Yuan, Li-dao Bao, Leng-ge Si

**Affiliations:** 1 Department of Pharmacy, Affiliated Hospital of Inner Mongolia Medical University, Hohhot, 010059 Inner Mongolia People’s Republic of China; 2 Department of Pathology, Affiliated Hospital of Inner Mongolia Medical University, Hohhot, 010059 Inner Mongolia People’s Republic of China; 3 Mongolia Medical School, Inner Mongolia Medical University, Hohhot, 010110 Inner Mongolia People’s Republic of China

**Keywords:** Piperine, Network pharmacology, Sciatica, PPARG, NF-kB1, Molecular docking

## Abstract

**Abstract:**

Piperine is the main active component of *Piper longum* L., which is also the main component of anti-sciatica Mongolian medicine Naru Sanwei pill. It has many pharmacological activities such as anti-inflammatory and immune regulation. This paper aims to preliminarily explore the potential mechanism of piperine in the treatment of sciatica through network pharmacology and molecular docking. TCMSP, ETCM database and literature mining were used to collect the active compounds of *Piper longum* L. Swiss TargetPrediction and SuperPred server were used to find the targets of compounds. At the same time, CTD database was used to collect the targets of sciatica. Then the above targets were compared and analyzed to select the targets of anti-sciatica in *Piper longum* L. The Go (gene ontology) annotation and KEGG pathway of the targets were enriched and analyzed by Metascape database platform. The molecular docking between the effective components and the targets was verified by Autodock. After that, the sciatica model of rats was established and treated with piperine. The expression level of inflammatory factors and proteins in the serum and tissues of rat sciatic nerve were detected by ELISA and Western blot. HE staining and immunohistochemistry were carried out on the sciatica tissues of rats. The results showed that *Piper longum* L. can regulate the development of sciatica and affect the expressions of PPARG and NF-kB1 through its active ingredient piperine, and there is endogenous interaction between PPARG and NF-kB1.

**Graphic abstract:**

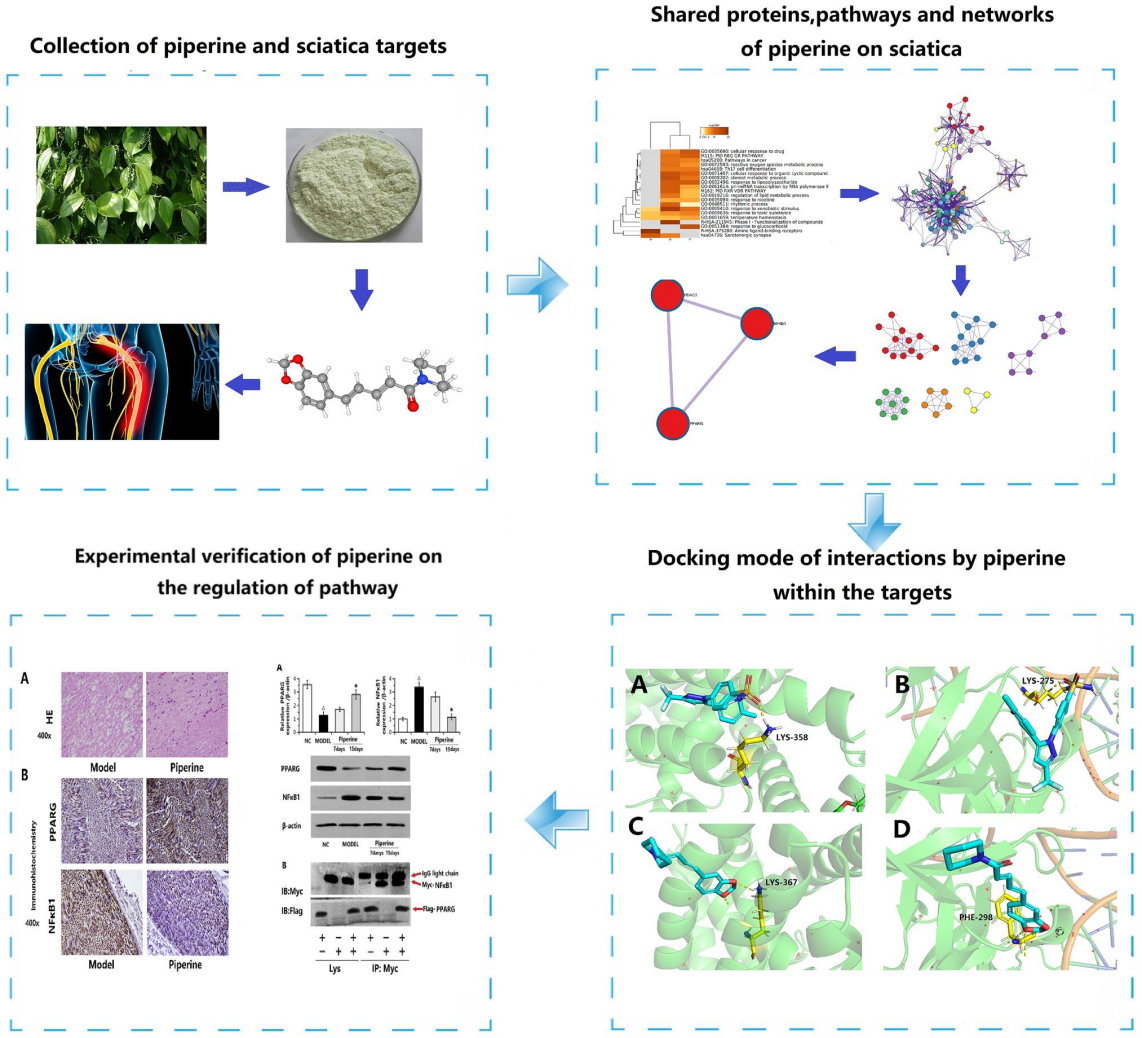

## Introduction

Sciatica is a kind of combined pain caused by stimulation and compression of various factors, such as lumbar disk herniation, lumbar degenerative disease and sciatic neuritis, which leads to the pain of stabbing, burning and blunt pain along the line of sciatica nerve walking, as well as the surrounding area, and brings great physiological and psychological pain to patients [1–3]. The mechanism of sciatica is very complex, which is often caused by the protrusion of lumbar disk [4]. Lumbar disk herniation, which can lead to injury of spinal nerve root and autoimmune inflammatory reaction of nucleus pulposus, is the mechanism of sciatica [5]. At present, the treatment of medicine can be divided into drug treatment and non-drug treatment. Among them, the main drug-based therapies are analgesics, such as ibuprofen, celecoxib, patazoxin and glucocorticoids, but these two kinds of drugs are prone to related side effects [6, 7]. Surgical treatment is also a common method, that is, decompression of spinal nerve root, so that patients are no longer subject to compression caused by lumbar disk herniation. But the operation can only delay the pain, and the treatment for the patients who have been sick for more than 2 years is not significant. After operation, infection and cerebrospinal fluid leakage are easy to occur [8]. There is no diseases’ name of lumbar disk herniation and sciatica in classic books of Mongolian medicine, but their causes of disease and clinical symptoms are similar to “lower limb’s Baimai disease” described in Mongolian medicine. Therefore, in Mongolian medicine, sciatica is classified as “lower limb’s Baimai disease.” In the early stage, the Mongolian people were nomadic, living in a very cold and humid environment, and the herdsmen who were used to riding were easy to get wet and fall to the ground. These environmental factors and trauma can lead to “Heyi” combating blood in Baimai and the long-term disease will lead to the excessive “Badagan,” resulting in the imbalance of three roots (Heyi, Badagan, Blood), thus damaging the Baimai of the lower limbs and causing the disease. Therefore, improving the blood circulation of Heyi, eliminating Badagan, regulating the body hormone and relieving pain are the main treatment principle [9].

Naru Sanwei Pill (Naru-3) is contained in the “National Medicine Standards of the Ministry of Health of the People’s Republic of China” (Mongolian Medicines). Its prescription is from Supreme and Important Prescription and consists of such three medicines as Radix aconiti agrestis, Fructus chebulae and *Piper longum* L [10]. In the prescription, Radix aconiti agrestis can eliminate “sticky,” relieve pain and dry dampness; Fructus chebulae can strengthen and detoxify; *Piper longum* L. can adjust the balance of the three roots (Heyi, Badagan, Blood), which has the effects of warming, dispersing cold and dehumidification. The combination of three Mongolian medicines in Naru-3 has the effect of adjusting Badachan and Heyi, regulating the balance of three roots and then treating rheumatism, joint pain and sciatica. The medicine is simple in composition, reasonable in formula and accurate in curative effect [11]. Piperine is a kind of monomeric compound extracted from black pepper plants and an alkaloid belonging to cinnamamide [12]. It is rich in Mongolian medicine *Piper longum* L. and has a wide range of pharmacological activities. At this stage, it has been found that piperine has good biological functions such as anti-oxidation, immune regulation and anti-inflammatory [13–15]. It has great potential in the treatment of osteoarthritis and sciatica. Existing pharmacological studies have shown that piperine can inhibit the inflammatory response and bone destruction of sciatic nerve. However, the mechanism of its anti-sciatica effect has not been fully elucidated [16].

The computer simulation research uses data mining, reverse molecular docking, computer simulation modeling and other technologies to study the biological network relationship among drugs, targets and diseases from the perspective of system biology and multidirectional pharmacology, and to systematically clarify the pharmacological mechanism of drugs, which is a new drug research method [17]. Zheng et al. used supercritical CO_2_ to extract the effective components of rongjinniantong formula, and successfully clarified the material basis, target and characteristics of rongjinniantong formula in the treatment of sciatica through computer simulation [18]. Yan et al. studied the material basis and molecular mechanism of Tripterygium Wilfordii in the treatment of sciatica based on computer simulation and confirmed the prediction by Western blotting and other technologies [19]. Many researches showed that computer simulation research can effectively predict the pharmacological mechanism of multi-component and multi-target action in traditional Chinese medicine. Therefore, this study used the combination of network pharmacology, molecular docking and experimental verification to provide new ideas and methods for anti-sciatica activity/mechanism of action.

## Materials and methods

### Collection and screening of candidate active compounds in *Piper longum* L.

TCMSP (http://lsp.nwu.edu.cn/tcmsp.php) and ETCM (http://www.nrc.ac.cn:9090/ETCM/index.PHP/home/index/) were searched to collect the related active compounds of *Piper longum* L. [20, 21]. TCMSP database screening criteria: oral bioavailability (OB) ≥ 30% and drug-likeness (DL) ≥ 0.18; ETCM database screening principles are five principles of Lipinski drugs (molecular weight less than 500; number of hydrogen bond donors less than 5; number of hydrogen bond acceptors less than 10; lipid water partition coefficient less than 5; number of rotatable bonds no more than 10) [22]. The molecular structure of each active compound was confirmed by TCMSP database and PubChem database (https://pubchem.ncbi.nlm.nih.gov/).

### Prediction of potential targets

Using the PubChem database, all compounds were transformed into the standard canonical smiles format, and the smiles format files were imported into Swiss TargetPrediction (http://www.swisstargetprediction.ch/) and SuperPred website (http://prediction.charite.de/), and the attribute was set to “home sapiens” to predict the targets of compounds. Swiss TargetPrediction selected the targets whose probability is ≥ 0.6 in the prediction results for further analysis. SuperPred can predict the potential targets of unknown molecules by calculating the Tanimoto similarity between molecules and more than 300,000 known compounds in the server. These known compounds include biological drugs, chemical drugs, experimental drugs, etc. The prediction results of Swiss TargetPrediction and SuperPred are collected and removed duplicates, which are used as the prediction targets of *Piper longum* L, so as to make further analysis [23, 24].

### Collection of targets related to sciatica

Selected a target with an Inference Score of 10 or higher from the CTD database (http://ctd.Mdibl.Org/) as a target for anti-sciatica drugs and established the sciatica-related target database in combination with the literature mining methods [25]. By comparing and analyzing the common targets of *Piper longum* L. and the related targets of sciatica, the prediction targets with definite anti-sciatica effect were concluded, and the gene library of anti-sciatica targets of *Piper longum* L. was finally established.

### Protein and gene information correction

Because the retrieved targets may have irregular names, contain proteins and genes from different species, the target information needs to be standardized after each step of target collection. The specific methods are as follows: UniprotKB search function is adopted in UniProt database (https://www.uniprot.org/), and protein name is input and restricted to human (Homo sapiens). All retrieved proteins are corrected to official symbol and standard gene names are extracted, and correct target information is obtained through data base search and transformation [26].

### Gene analysis and pathway annotation

The Metascape platform (http://metascape.org) is a gene annotation analysis database, which is used to analyze the biological processes and pathways of genes [27]. The targets of *Piper longum* L. against sciatica were inputted to the Metascape platform. After submitting, the attributes were set to “home sapiens” and *P* < 0.01. GO annotation analysis and KEGG pathway analysis on the targets were performed, and the results were saved and sorted by the number of targets involved in each entry to screen top biological processes and pathways.

### Functional attribution of target protein

Metascape platform for gene annotation analysis can not only analyze the biological process and pathway enrichment of the input gene, but also map the gene corresponding to the regulatory target protein of *Piper longum* L. directly to the pathway [28]. The pathway enriched by the drug target is considered as the drug regulatory pathway. From the network diagram of compound-target-pathway, we analyzed the common targets of *Piper longum* L. against sciatica and defined the synergism or superposition between drugs and pathways.

### Molecular docking verification

Through the above-mentioned Metascape analysis, the important signal pathway/key targets and main active components for the treatment of sciatica were obtained. The key target in the important signal pathway and the main active component in *Piper longum* L. were linked by molecular docking. Use the RCSB PDB database (http://www.rcsb.org/pdb/home/home.do) to retrieve and download 3D structure files for key target proteins while utilizing the ZINC database (http://zinc.docking.org/) to download the 3D structure file of the active compound. Before docking, the ligands and acceptors need to minimize energy, delete the water molecules of acceptors (PDB files), add polar hydrogen atoms, give charge and add magnetic field. All substructures within the radius of 0.65 nm were used as the active pocket of the binding site [29]. All parameters are set by default except for special instructions. The Autodock Molecular Docking software (version 2.5) was used to validate the network pharmacology screening results by docking the active compound and the positive drug celecoxib with the receptor protein molecule [30]. The results were visualized by PyMOL software, and the hydrogen bonds and their binding sites were observed and analyzed. The docking energy value was determined by the consistency score function of ligand-receptor affinity. The purpose of this study is to examine the binding mode and binding free energy of the compound with the corresponding target to determine the affinity between them.

### Laboratory animals and groups

A total of 48 adult male SD rats, weighing 200–220 g (experimental animal center of Inner Mongolia Medical University, Hohhot, China), with room temperature of 20 ± 1 °C, humidity of 40-50%, 12 h of sunlight, were fed with standard rodent food and water. The rats were randomly divided into sham group, model group, celecoxib group and piperine group, with 12 rats in each group.

All experiments in the present study were approved by the Animal Experimental Ethics Committee of Inner Mongolia Medical University. The efforts were made to minimize the number of animals used in the studies. During the study, humane end points were used in accordance with Affiliated Hospital of Inner Mongolia Medical University standard operating protocol. In case of infection at the surgical site, wound dehiscence, weight loss (> 20%) or if the animal became cachectic had difficulty eating, drinking or moving around freely, the animal was euthanized by inhalation of carbon dioxide. No animals showed signs of peritonitis throughout the study. No animal deaths occurred throughout the experiment.

### A rat model of non-compression lumbar disk herniation and evaluation of pain-related behaviors

Rats were anaesthetized by injection of 10% chloral hydrate into their abdominal cavity according to a dose of 3.5 ml/kg. With the L4-L6 spinous process as the center, the longitudinal incision of the midline back was performed to expose the bilateral L4 and L5 lamina. The two intervertebral disks of the larger tail were found along the lumbar vertebrae, removed the nucleus pulposus 5 mg and then gently placed the nucleus pulposus at the ruptured ganglion and finally sutured. The sham group sutured directly after destroying the ganglion and did not place the nucleus pulposus. Mechanical withdrawal threshold (MWT) of four groups was measured, respectively, before the operation, at the first, third, fifth, seventh, tenth and fourteenth day after the operation and pain-related behavior evaluation was performed to determine whether the model was established successfully [31].

### Dosage and method

Sham group: no oral administration. Model group: 0.75 ml normal saline was given to the stomach every day. Celecoxib group: celecoxib (Pfizer, California, USA, J20140072) was prepared into suspension with 0.9% normal saline and administered by gavage 0.75 ml every day. Piperine group: piperine (purity > 97%, Sigma, Missouri, USA) be dissolved by purified water and made into suspension, 0.75 mL of which was given to rats by intragastric administration every day. All the above drugs were given for 15 consecutive days.

### Detection of inflammatory factors in rats

The serum levels of pro-inflammatory factors (IL-1β, TNF-α) and anti-inflammatory factors (IL-10, TGF-β1) were measured by enzyme-linked immunosorbent assay (ELISA) [32, 33].

From the 15th day of administration, the rats in each group were anesthetized by intraperitoneal injection of 10% chloral hydrate (3 ml/kg). After a few minutes, the rats were gently clamped on their toes with tweezers, if there were no reactions, the rats were judged to have entered a deep anesthesia state. Then, the rats were killed by cervical dislocation as quickly as possible. When the rats stopped breathing, the blood was collected from the abdominal aorta, centrifuged at 3000 r/min for 15 min, and the supernatant was stored at − 80 °C for ELISA and biochemical detection. The sciatic nerve tissue of rats was taken out in sterile condition with sterilized instruments, and the skin was peeled off, and placed at − 80 °C for Western blot detection.

### He staining and immunohistochemical analysis of sciatic nerve tissue in rats

First, the tissue sections were made, and the thin sections were put into warm water of 50 °C, loaded with glass slides, and then dried at 38 °C for 2 h. The dried tissue sections were dewaxed and hydrated. The antigen repair and sealing of the tissue sections were carried out by boiling the citrate tissue antigen repair solution directly. The tissue sections were placed in the prepared repair solution, placed in the microwave oven and heated for 5 min at high temperature for three times. Then the tissue slices were taken out, cooled naturally for 10 min, washed with tap water for 3 min, three times. Finally, it was rinsed by PBS solution for 3 min, three times. PBS solution was removed, Rabbit anti-human NF-kB1 (Cell Signaling Technology, Inc. USA) and PPARG gene polyclonal antibodies (NeuroMab,Inc. USA) were added and incubated overnight at room temperature. Enzyme labeled Goat anti-rabbit IgG polymer antibody (Santa Cruz Biotechnology, USA) was added, incubated at 37 °C for 15 min, DAB color solution was added, incubated for 5 min. After the counterstaining, the sections were dehydrated, transparent and finally sealed with a neutral gum and a cover slip. The dewaxed and hydrated tissue sections were stained with hematoxylin for 5.5 min, washed with tap water for 10 s, then returned to blue and finally stained in eosin for 8 s. The stained sections were dehydrated and transparent and then sealed with a neutral gum and a cover glass.

### Western blotting for PPARG and NF-kB1 expression

The sciatic nerve tissue of each group was collected, the tissue cells were lysed, and the supernatant of cytoplasmic and nuclear proteins were extracted. After electrophoresis for 2 h, the method of wet transfer was used. It was transferred to PVDF membrane by electric transfer. In the closed solution of 5% skimmed milk TBST, the TBST was rinsed at room temperature for 1 h and then added with primary antibodies. It was shaken overnight at 4 °C and rinsed several times by TBST solution. Polyclonal antibody of Rabbit anti-human-related proteins were added and rinsed three times after shaking at room temperature for 1 h to detect the levels of PPARG and NF-kB1.

### Immunocoprecipitation test

Human sciatic nerve cells (Shanghai Hongshun Biotechnology Co., Ltd. China) were cultured in RPMI1640 medium containing 10% BCS and subcultured into a 10-cm culture dish. The PPARG plasmid (pcDNA3.0-flag-PPARG) and NF-kB1 plasmid (pCMV-Myc-NF-kB1,Guangzhou Ruibo Biotechnology Co., Ltd, China) were co-transfected into human sciatic nerve cells with the Supeffect transfection reagent. The cells were harvested, and the total protein was extracted after 48 h. The protein supernatant was mixed with 2 μg antibody anti-myc and incubated at 4 °C for 3 h with gentle shaking. The protein A/G agarose beads were added for 40 μL and incubated at 4 °C for 8 h with gentle shaking. The beads were centrifuged at 4 °C and eluted with lysate for three times. The sample buffer of 2 × SDS was added. The sample was treated at 100 °C and added with 10% SDS-PAGE glue. After electrophoresis and membrane transfer, Western blot was carried out with flag antibody to detect whether there was Flag-PPARG in the complex. NF-kB1 (pCMV-Myc-NF-kB1) and PPARG (pcDNA3.0-flag-PPARG) were used as control group.

### Data analysis

All data are expressed as average numbers ± SD. SPSS 20.0 has been used for the statistical analysis. Differences between groups were determined by Student’s *t* test and analysis of variance of repeated measures. *P* < 0.05 was considered statistically significant, and there was a difference. *P* < 0.01 means significant difference.

## Result

### Collection and screening of candidate active compounds in Piper longum L

**Fig. 1:**
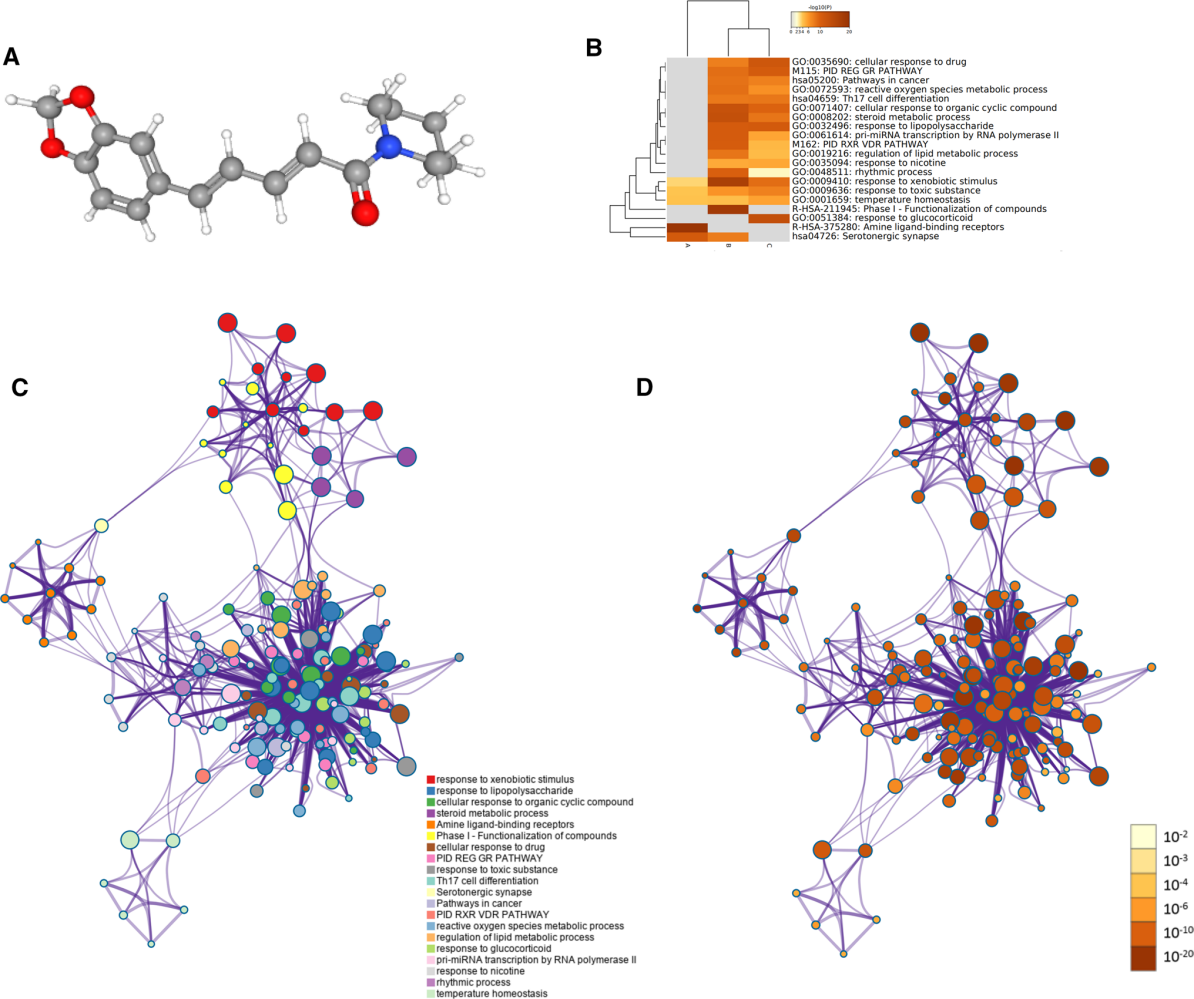
**a** The 3D structure of piperine; **b** the Metascape platform to select the top GO annotation results and KEGG pathway; **c** the map of differential gene enrichment interaction, including 20 groups of enrichment results; **d** the network map constructed according to the enrichment degree, the darker the color is, the more genes are enriched into the pathway

On the basis of TCMSP and ETCM, the molecular structure of each active compound was confirmed by TCMSP and PubChem database. At the same time, according to oral bioavailability, drug similarity and Lipinski principle, nine effective compounds of *Piper longum* L. were screened and sorted out, piperine was selected as the candidate drug (Fig. 1a). Table 1 is the information of *Piper longum* L. active compounds screened from ETCM database, and Table 2 is the information of *Piper longum* L. active compounds screened from TCMSP database.

**Table 1:** Information on active compounds of *Piper longum* L. screened by ETCM

Number	Compound name	Chemical formula	Molecular weight	Number of hydrogen bond donors	Number of hydrogen bond acceptors	ALogP	Number of rotatable bonds
1	Pipercide	C22H29NO3	355.47	1	3	5.226	10
2	Laurotetanine	C19H21NO4	327.37	2	5	2.772	3
3	Piplartine	C17H19NO5	317.34	0	5	2.135	5
4	Piperlonguminine	C16H19NO3	273.32	1	3	2.933	5
5	Piperine	C17H19NO3	285.33	0	3	2.863	3
6	Sesamin	C20H18O6	354.35	0	6	2.237	2
7	Tetrahydropiperic acid	C12H14O4	222.23	1	4	2.631	5

**Table 2:** Information on active compounds of *Piper longum* L. screened by TCMSP

Number	Compound name	Chemical formula	OB/%	DL
1	Sesamin	C20H18O6	56.55	0.83
2	Piperlonguminine	C16H19NO3	30.71	0.18
3	*N*-(2,5-dimethoxyphenyl)-4-methoxybenzamide	C16H17NO4	60.7	0.18
4	*N*-Isobutyl-2,4-icosadienamide	C24H45NO	38.86	0.32
5	Piperine	C17H19NO3	42.52	0.23

### Target prediction

Target prediction of *Piper longum* L. active compounds was carried out according to Swiss and SuperPred Web site. There were 116 putative targets in nine active compounds of *Piper longum* L, and 70 after removed duplicates. According to the inference score, 55 known therapeutic targets of sciatica in CTD database were selected to construct the target database of sciatica.

### Gene function and pathway enrichment analysis

Using Metascape platform, Go annotation analysis and KEGG pathway analysis of potential targets of *Piper longum* L. against sciatica were carried out, and the threshold value *P* < 0.01 was set to screen the top GO annotation results and KEGG pathway. The results are shown in Fig. 1b. The results showed that there were 20 highly overlapped signaling pathways, among which KEGG pathway related to inflammation was selected to map the gene corresponding to the regulatory target protein of *Piper longum* L. directly to the pathway. Through the enrichment analysis of KEGG pathway in the Metascape database, three pathways closely related to inflammation are mainly nuclear factor kappa B (NF-kB) signaling pathway, Fc epsilon RI signaling pathway and inflammation mediator regulation of TRP channels. To further determine the relationship between enriched terms, Kappa scores were calculated as a measure of similarity between terms, and an enriched term similarity network was constructed, as shown in Fig. 1c. Kappa score is an indicator of the extent to which two raters who are examining the same set of categorical data, agree and takes into account agreement occurring by chance [34]. The nodes are connected to form a network by the similarity between terms (Kappa > 0.3), and each node represents an enrichment term. The color of the node in Fig. 1c indicates the cluster to which the node belongs. It can be seen that the terms belonging to the same cluster are closer and more closely related to each other. The color of the nodes in Fig. 1d indicates the degree of enrichment (*P* value), and it can be seen that the more the number of genes is included, the more significant the *P* value is.

### Functional attribution of target protein

**Fig. 2:**
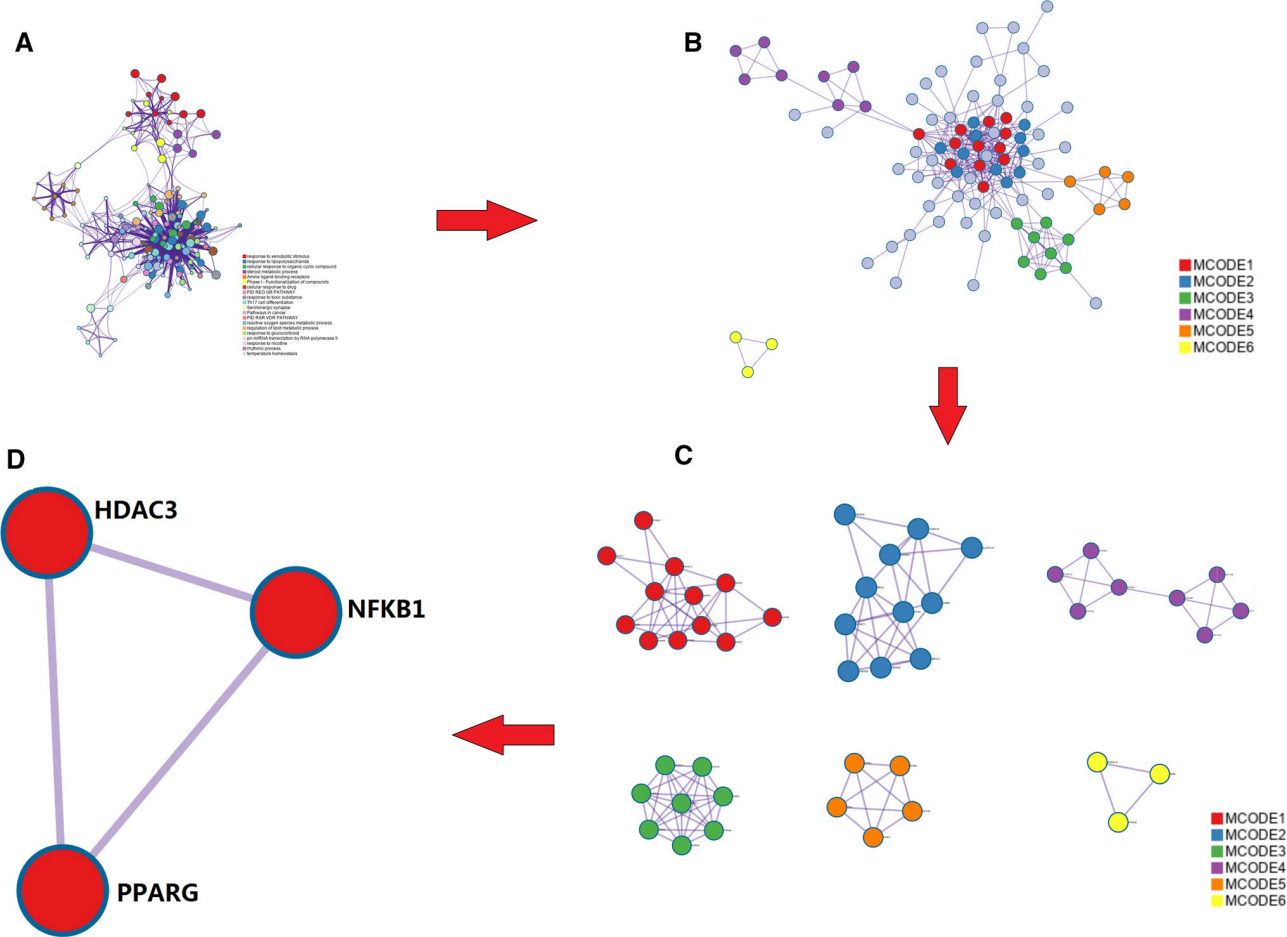
**a** The fully connected interaction network of all gene-related proteins; **b** the module substructure recognized in the interaction network; **c** the modules are abstracted from the fully connected interactive network. **d** The key gene corresponding to the target protein on the pathway

Through the Metascape platform, all genes are connected to the whole protein interaction network (Fig. 2a). Six different colors represent the module substructure (Fig. 2b) recognized in the interaction network. Figure 2c is formed by abstracting the modules from the fully connected interaction network. The key genes corresponding to the regulatory target protein of *Piper longum* L. were mapped directly to the pathway (Fig. 2d). The pathway enriched by the drug targets was considered as the drug regulatory pathway. It can be found that on the NF-kappaB signaling pathway and the inflammatory mediator regulation of TRP channels, three target proteins PPARG, NR3C1 and NF-kB1 are found to have mutual relationship, among which NF-kB1 and PPARG are key targets related to inflammation, and their corresponding compound is piperine.

### Results of piperine target molecule docking

As a non-steroidal anti-inflammatory drug, celecoxib is often used to treat osteoarthritis, relieve the symptoms of adult sciatica and treat adult acute pain [35]. Therefore, celecoxib is used as a positive drug for molecular docking.

**Fig. 3:**
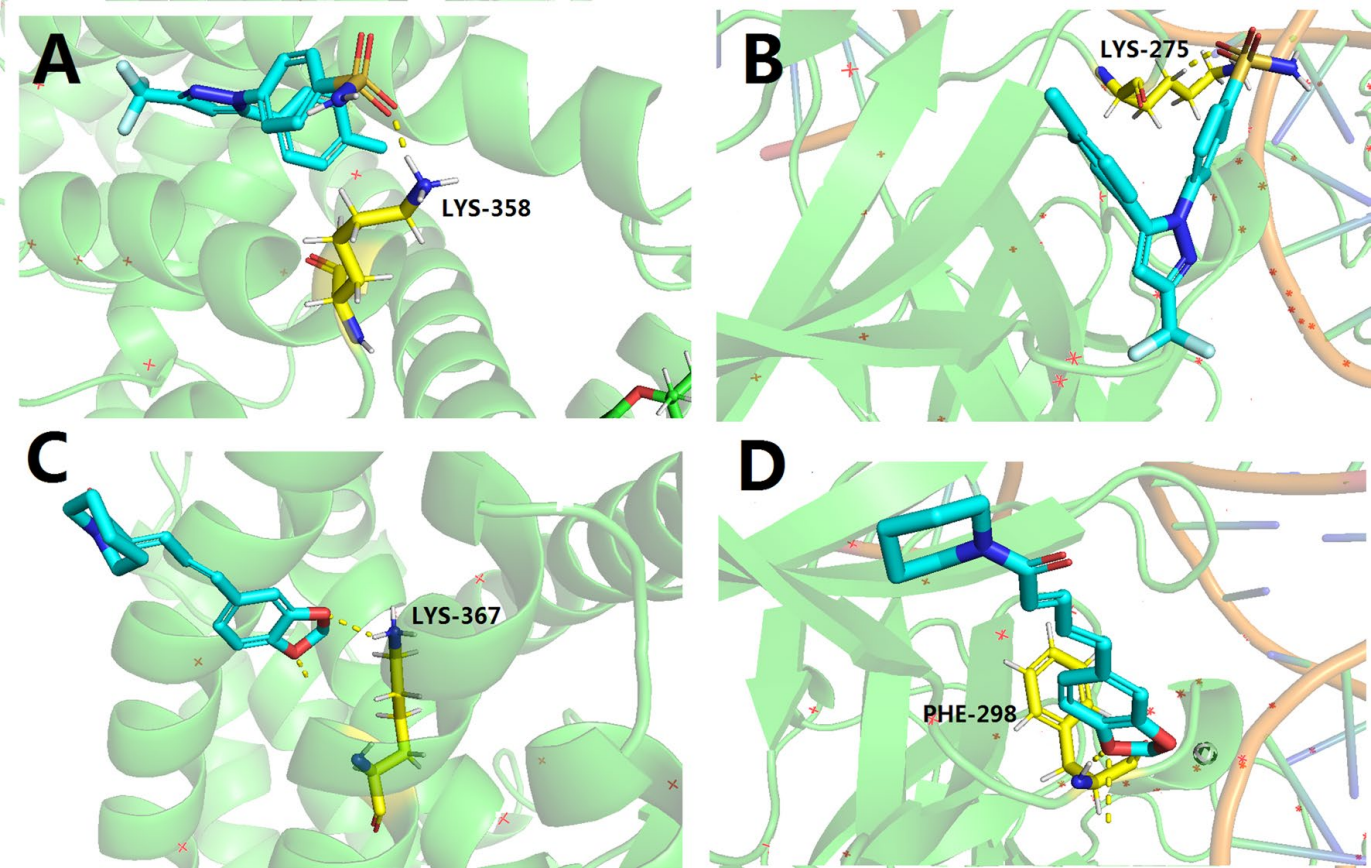
**a** The site binding of celecoxib and PPARG. The blue is the 3D structure of celecoxib, and the yellow is LYS-358, the amino acid residue of PPARG; **b** the site binding of celecoxib and NF-kB1. The blue is the celecoxib, and the yellow is LYS-275, the amino acid residue of NF-kB1; **c** the site binding of piperine and PPARG. The blue is the 3D structure of piperine, and the yellow is LYS-367, the amino acid residue of PPARG; **d** the site binding of piperine and NF-kB1. The blue is the piperine, and the yellow is PHE-298, the amino acid residue of NF-kB1

Based on the above screening results, PPARG and NF-kB1 were verified. The 3D structure is imported into Autodock and docked with piperine and celecoxib, respectively. Their interaction mode with key amino acids and their binding at the active site are shown in Fig. 3. The energy values of the compounds shown in the docking results are shown in Table 3. The molecular docking results show that compared with the commercialized small molecular inhibitors, the core components and targets have better binding activity. Piperine has a good docking with the corresponding protein, and the key amino acids around it mainly play a role in the form of hydrogen bonds. The formation of hydrogen bonds minimizes the energy of the small molecule–receptor complex and is therefore the most stable. Combined with Table 3, the docking energy value is relatively small, indicating that the compound can stably bind to the receptor protein and play a role. The results also indirectly showed that the molecular docking results are consistent with the results of network pharmacology screening, which further verifies the reliability of network pharmacology prediction target. However, compared to piperine, the docking energy of celecoxib is smaller, indicating that celecoxib can bind to receptor proteins more stably and the effect of piperine on molecular docking anastomosis is not as good as celecoxib (Table 4).

**Table 3:** Binding affinity of piperine and celecoxib in the treatment of sciatica via molecular docking

Chemical compound	Putative target	PDB ID	Binding energy (kcal/mol)
Piperine	PPARG	6C5Q	− 4.01
Piperine	NF-kB1	1OOA	− 3.72
celecoxib	PPARG	6C5Q	− 1.36
celecoxib	NF-kB1	1OOA	− 2.63

**Table 4:** Results of preoperative and postoperative MWT values in four groups of rats ($\overline{x} \pm {\text{SD}}$)

Group	MWT (g)			
	Sham	Model	Celecoxib	Piperine
Preoperative	35.13 ± 3.28	34.62 ± 2.35	35.96 ± 2.90	33.93 ± 3.58
1 day after surgery	26.41 ± 3.63	18.70 ± 1.44	19.80 ± 2.05	19.19 ± 2.40
3 days after surgery	24.63 ± 3.16	16.46 ± 0.93**	17.68 ± 2.27*	18.53 ± 2.06
5 days after surgery	28.29 ± 2.64	15.21 ± 1.08**	15.75 ± 1.83*	16.66 ± 3.24
7 days after surgery	29.48 ± 2.46	9.96 ± 3.19**	11.70 ± 2.37**	10.33 ± 1.70**
10 days after surgery	31.31 ± 2.96	9.28 ± 2.35	10.06 ± 1.63	8.55 ± 1.22**
14 days after surgery	33.64 ± 1.95	7.56 ± 2.11	8.64 ± 1.85	6.75 ± 1.08**

The model group, celecoxib group and piperine group were compared with the sham group at each time point after surgery *P < 0.05; **P < 0.01

### The model of non-compression lumbar disk herniation in rats

By measuring the MWT values of rats in sham group, model group, celecoxib group and piperine group without drug administration, we found that there was no significant difference in the MWT values of the four groups before operation (*P* > 0.05); there was significant difference in the MWT values at each time point after operation and before operation (*P* < 0.01), indicating that the rats in the model group, celecoxib group and piperine group have developed pain. When the MWT values of the rats in model group, celecoxib group and piperine group were compared with each other, it was found that there was a significant difference between the 3 days and 1 day, 5 days and 3 days after surgery (*P* < 0.01), and the celecoxib group had difference (*P* < 0.05). There was no significant difference in the piperine group (*P* > 0.05). When compared with 5 days and 7 days after surgery, the MWT values of the rats in model group, celecoxib group and piperine group showed significant differences (*P* < 0.01), indicating that the rats showed pain peak from the 7th day after surgery. The results of this experiment are consistent with the reports, indicating that the rat model of this study was successfully produced.

### Expression of inflammatory factors in serum and tissues of rats

The levels of pro-inflammatory factors (IL-1β, TNF-α) and anti-inflammatory factors (IL-10, TGF-β1) in the serum of rats were measured by ELISA. It was found that the levels of IL-1β, TNF-α in the serum of rats were significantly higher than those of sham rats (*P* < 0.01), and the levels of IL-10, TGF-β1 were significantly lower (*P* < 0.01). Compared with the model rats, the contents of IL-1β and TNF-α in celecoxib group and piperine group decreased significantly (*P* < 0.01), while the contents of IL-10 and TGF-β1 increased significantly (*P* < 0.01). The results showed that the inflammatory response of rats was prominent before administration, the content of pro-inflammatory factors in serum increased, the content of anti-inflammatory factors decreased, but the inflammatory response was relieved after administration of celecoxib and piperine, so the content of IL-1β and TNF-α in serum decreased, and the content of IL-10 and TGF- β1 increased.

**Fig. 4:**
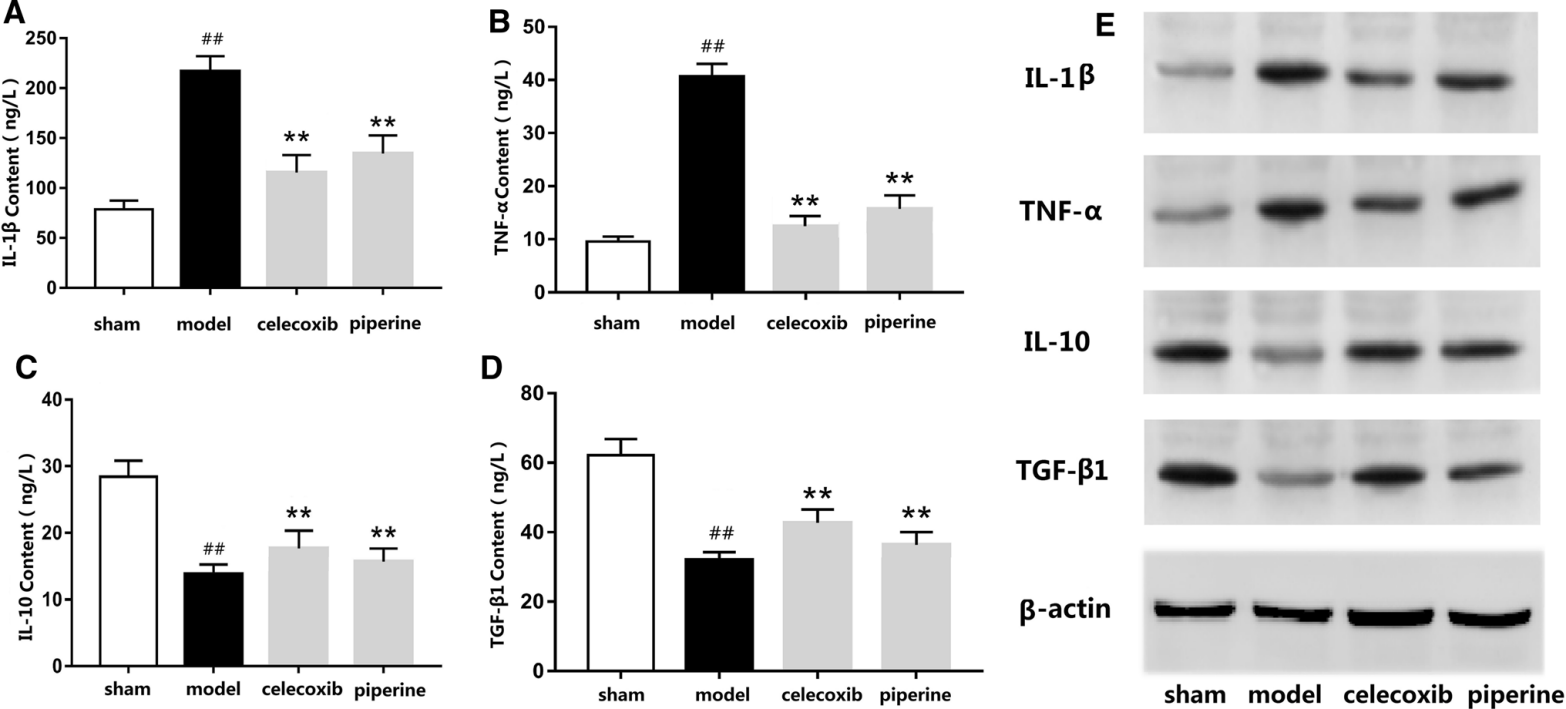
**a** The expressions of IL-1β, TNF-α, IL-10 and TGF-β1 at protein levels of rat sciatic nerve in four groups. **b**–**e** The contents of IL-1 β, TNF-α, IL-10 and TGF-β1 in serum of four groups. ##*P* < 0.01 compared with sham rats. **P < 0.01 compared with model rats

Western blot analysis was used to determine the protein expressions of various inflammatory factors in the sciatic nerve, as shown in Fig. 4a. Compared with sham rats, the expressions of IL-1β and TNF-α in the model rats increased significantly (*P* < 0.01), and the expressions of IL-10 and TGF-β1 decreased significantly (*P* < 0.01); the expression of IL-1β and TNF-ɑ was significantly decreased after administration (*P* < 0.01), and the expression of IL-10 was increased in the piperine group (*P* < 0.05). IL-10 in the celecoxib group and TGF-β1 in the two administration groups were significantly increased (*P* < 0.01). This result indicated that piperine can reduce the level of pro-inflammatory factors (IL-1β, TNF-ɑ) and increase the level of anti-inflammatory factors (IL-10, TGF-β1) at the protein level.

### Pathological analysis of sciatic nerve

**Fig. 5:**
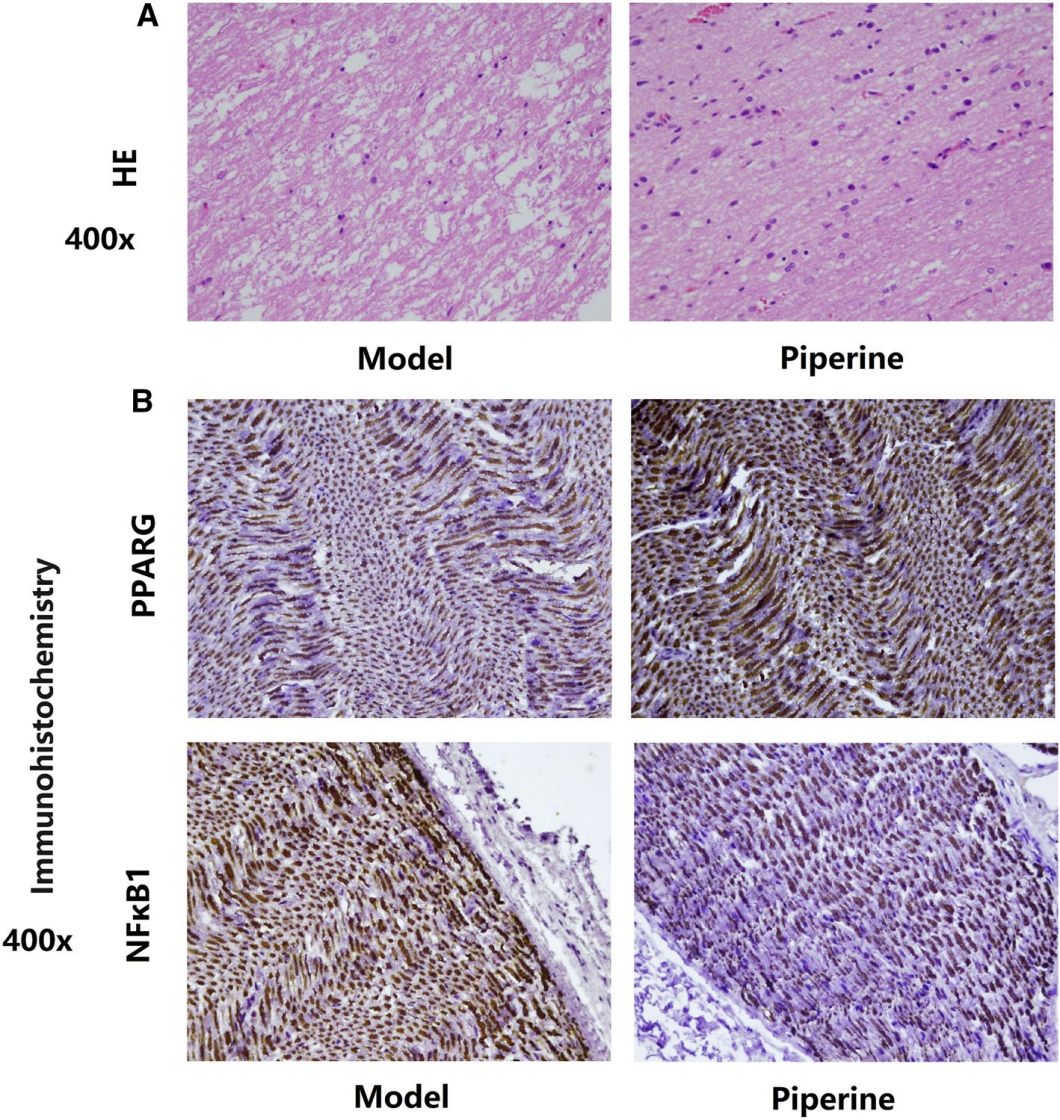
Pathological effects of piperine on sciatica model rats. **a** The HE staining results of the model group and piperine group, respectively. **b** The results of PPARG and NF-kB1 immunohistochemistry in the model group and piperine group, respectively, and the positive expression appeared in the cytoplasm

The histomorphology of sciatic nerve in rats before and after administration was analyzed by HE staining (Fig. 5a). We found that the arrangement of nerves in the sham group was tight and orderly. Compared with the sham rats, the degree of nerve damage in the model group was extremely severe, obvious vacuoles appeared, the nerve fibers were severely deformed, and light staining occurred. After administration, compared with the model group, the vacuoles in piperine group were significantly reduced, and the degree of nerve fiber damage was also improved. It is suggested that piperine has some effects on the repair of injured sciatic nerve. The results of immunohistochemistry showed that compared with the model group, the positive expression rate of PPARG protein in the sciatic nerve tissue in the piperine group was increased, with a statistically significant difference (*P* < 0.05); compared with the model group, the positive expression rate of NF-kB1 in the sciatic nerve tissue in the piperine group was decreased. Both proteins are expressed in the nucleus, and there is a possibility of co-expression. (Figure 5b).

### Western blot and immunocoprecipitation proved the relationship between PPARG and NF-kB1

**Fig. 6:**
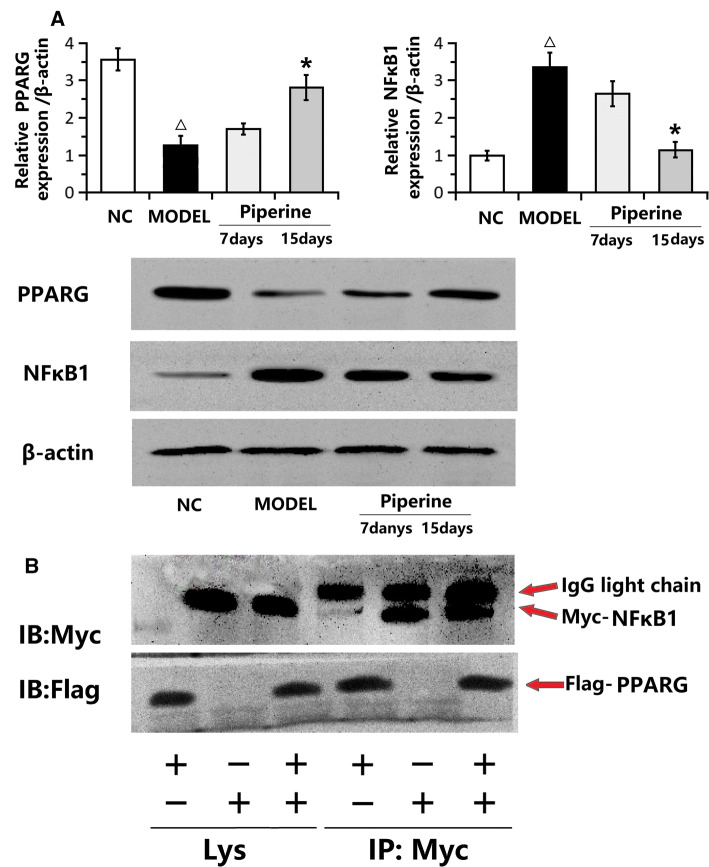
Western blot and immunocoprecipitation. **a** Western blot showed that NF-kB1 expression in piperine group was significantly lower at 15 days than that at 7 days (*P* < 0.01), and PPARG expression increased with piperine administration (*P* < 0.01). **b** Immunocoprecipitation showed that PPARG was detected in the experimental group, but not in the negative control group, indicating that NF-kB1 protein and PPARG protein can specifically bind

Western blot showed that NF-kB1 expression in piperine group was significantly lower at 15 days than that at 7 days (*P* < 0.01), and PPARG expression increased with piperine administration (*P* < 0.01) (Fig. 6a). In the immunocoprecipitation experiment, pcDNA3.0-flag-PPARG and pCMV-Myc-NF-kB1 plasmids were co-transfected into human sciatic nerve cells, immunocoprecipitation was performed with antibody Myc, and Western blot was performed with antibody flag. The results showed that PPARG was detected in the experimental group, but not in the negative control group (Fig. 6b). The above experiments were repeated for three times under the same conditions, and the same results were obtained. The results showed that NF-kB1 protein and PPARG protein can specifically bind, and the two proteins interact in cells.

## Discussion

Sciatica is a syndrome characterized by pain in the route and distribution of the sciatic nerve, with pain as the main performance. The common pain areas include the waist and buttocks, the back of the big/lower leg, the outside of the foot, etc., especially when the patient bends, coughs, sneezes and other actions, the pain is abnormal [36]. Naru-3 is made of the Radix aconiti agrestis, the Fructus chebulae and *Piper longum* L. It has the effects of agitation and detoxification, promoting blood circulation, regulating immunity, reducing swelling and relieving pain. It is increasingly used in the treatment of cervical spondylosis, lumbar disk herniation, sciatica and other diseases [37]. The main medicine in the prescription is *Piper longum* L. Piperine is the main active component of *Piper longum* L, which has many biological functions such as anti-inflammatory and immunity. However, the mechanism of piperine against sciatica has not been fully elucidated. In this study, we combined network pharmacology to analyze the targets of anti-sciatica in *Piper longum* L and used molecular docking virtual calculations and experiments to verify the potential molecular mechanism of piperine against sciatica.

Network pharmacology is a research strategy of drug multi-target and multi-path interaction [38]. It is a powerful tool for the modern research of Mongolian medicine, which starts from the integrity and systematization of the interaction between drug targets and diseases, uses computational approaches for modeling of multi-target activity, and studies the biological basis of drugs acting on the body. Abeijon et al. [39] applied hansch model in theoretical–experimental studies of organic synthesis, pharmacological assay and prediction of unmeasured results for a series of compounds similar to Rasagiline (compound of reference) with potential neuroprotection effect. Speck-Planche et al. designed a multi-target inhibitor for breast cancer-related proteins by using fragment-based computer simulation [40]. Firstly, based on the strategy of network pharmacology, the interaction network of known sciatica-related genes was constructed, and the key candidate targets of intervention of *Piper longum* L. on sciatica-related imbalance network were selected. It was found that the key targets of *Piper longum* L. were significantly enriched in several pathways related to the pathological process of sciatica, among which NF-kB signaling pathway, a member protein of this signaling pathway, especially PPARG and NF-kB1, were closely related to the inflammatory response in the pathological process of sciatica [41].

PPARG is the gene encoding peroxisome proliferative activated receptor γ, and the protein encoded by PPARG belongs to the nuclear receptor peroxisome proliferative activated receptor subfamily [42]. PPAR family members can join with vitamin A receptor (RXR) to form heterodimers, which can regulate the transcription of many genes by binding to specific DNA sequences [43]. PPARG plays a fundamental role in immune response by inhibiting the expression of inflammatory cytokines and guiding the differentiation of immune cells to anti-inflammatory phenotype [44]. In recent years, PPARG has been found to regulate fat metabolism, inflammation, immunity and cell differentiation and participate in the occurrence and development of many chronic immune diseases [45]. NF-kB1 is a protein factor that can specifically bind to the enhancer KB sequence of immunoglobulin K light chain gene [46]. It can specifically bind to a variety of cell gene promoters or enhancer sequence-specific sites to promote transcription and expression, and participate in inflammatory response, immune response, cell proliferation, transformation and apoptosis and other important pathophysiological processes [47]. In the process of inflammation, NF-kB enters the nucleus after being activated, which has a strong ability to bind to the NF-kB1 gene site, and is the premise and key of transcription and release of cytokines. Moreover, most of the promoters or enhancers of cytokines (such as TNF-α, IL-1β, IL-6 and IL-8) have NF-kB binding sites, so the activated NF-kB entering the nucleus can combine with them and promote the transcription of these cytokines, thus causing inflammation [48]. Scirpor et al. found that PPARG could inhibit NF-kB signaling pathway and then reduce the expression of CXC chemokine, monocyte chemoattractant protein-1 and macrophage inflammatory protein-2 in the mouse model of cystic fibrosis [49]. Kim et al. found that rosiglitazone can activate PPARG, inhibit NF-kB signal pathway and then down-regulate the expression of IL-17, and reduce the airway inflammatory response in allergic airway inflammation model of mice [50]. Nakajima et al. found that PPARG gene deletion aggravates intestinal inflammation through NF-kB signaling pathway in mice with intestinal ischemia/reperfusion injury [51]. Tang et al. found that Rosmarin inhibited NF-kB signal pathway by up-regulating PPARG and reduced the expression of IL-6, TNF-α and CRP in MI of rats [52]. These studies suggest that PPARG can inhibit the development of inflammatory response by inhibiting NF-kB signaling pathway.

On the basis of the targets of anti-sciatica of *Piper longum* L. discovered by network pharmacology, this study used molecular docking virtual computing to explore the binding ability of piperine with related proteins. Molecular docking is a computational tool for predicting the binding ability and binding mode of proteins and ligands [53]. Its principle is based on the “lock key model” of the interaction between proteins and small ligands, calculating and predicting the conformation and orientation of ligands at protein active sites, so as to judge the binding degree and play an important role in the target prediction of drug organisms [54]. Autodock is a common molecular docking method. Autodock is developed by Scripps Research Institute. It uses Lamarckian genetic algorithm and simulated annealing to find the best binding position between protein and ligand and evaluates the docking result according to semi-empirical free energy function. It takes a long time, but the precision is very high [55]. In this study, using Autodock molecular docking tools, we found that piperine has a high affinity with PPARG and NF-kB1 protein in NF-kB signaling pathway (binding energy is less than—5.0). This method further proves the accuracy and reliability of the virtual computation of network pharmacology molecules and molecular docking. Based on the experimental verification of rat model, piperine can significantly improve the severity of sciatic nerve. In the model of sciatica rats treated with piperine, the expression of NF-kB1 was down-regulated, and the expression of PPARG was up-regulated. Therefore, we speculated that PPARG might reverse regulate the expression of NF-kB1 gene, which was confirmed to be directly regulated by immunocoprecipitation. Based on the above bioinformatics screening and preliminary experiments, it is confirmed that the direct regulatory relationship between PPARG and NF-kB1 may be the biological mechanism of the occurrence and development of sciatica, but its upstream control genes and downstream effector molecules are not very clear at present, which needs further study.

## Conclusion

In this study, network pharmacology analysis, molecular docking virtual computing and experimental verification of rat model based on non-compression lumbar disk herniation were used. It was found that piperine could significantly improve the severity of sciatica in rats. The targets were PPARG and NF-kB1, which were reverse regulatory relationship. PPARG can reduce inflammation by inhibiting NF-kB signal pathway. The results will provide theoretical basis for the pharmacological effects of *Piper longum* L. and piperine on sciatica and also provide methodological reference for the mechanism of anti-inflammatory active components of traditional Chinese medicine.

## Data Availability

The datasets used and/or analyzed during the current study are available from the corresponding author on reasonable request.

## Abbreviations

TCMSP: Traditional Chinese medicine systems pharmacology database and analysis platform
ETCM: The encyclopedia of traditional Chinese medicine
CTD: Comparative toxicogenomics database
PPARG: Peroxisome proliferative activated receptor, gamma
NF-kB1: Nuclear factor kappa B subunit 1
TRP: Transient receptor potential
NR3C1: Nuclear receptor subfamily 3 group C member 1
PVDF: Polyvinylidene fluoride
ELISA: Enzyme-linked immunosorbent assay
SD: Standard deviations
PBS: Phosphate-buffered saline
HE: Hematoxylin-eosin
DAB: Diaminobenzidine
NF-KB: Nuclear factor kB
MWT: Mechanical withdrawal threshold
ECL: Electrochemiluminescence
TBST: Tris-buffered saline tween
CRP: C-reaction protein
MI: Myocardial Infarction
